# Extraction of Substance Use Information From Clinical Notes: Generative Pretrained Transformer–Based Investigation

**DOI:** 10.2196/56243

**Published:** 2024-08-19

**Authors:** Fatemeh Shah-Mohammadi, Joseph Finkelstein

**Affiliations:** 1 Department of Biomedical Informatics School of Medicine The University of Utah Salt Lake City, UT United States

**Keywords:** substance use, natural language processing, GPT, prompt engineering, zero-shot learning, few-shot learning

## Abstract

**Background:**

Understanding the multifaceted nature of health outcomes requires a comprehensive examination of the social, economic, and environmental determinants that shape individual well-being. Among these determinants, behavioral factors play a crucial role, particularly the consumption patterns of psychoactive substances, which have important implications on public health. The Global Burden of Disease Study shows a growing impact in disability-adjusted life years due to substance use. The successful identification of patients’ substance use information equips clinical care teams to address substance-related issues more effectively, enabling targeted support and ultimately improving patient outcomes.

**Objective:**

Traditional natural language processing methods face limitations in accurately parsing diverse clinical language associated with substance use. Large language models offer promise in overcoming these challenges by adapting to diverse language patterns. This study investigates the application of the generative pretrained transformer (GPT) model in specific GPT-3.5 for extracting tobacco, alcohol, and substance use information from patient discharge summaries in zero-shot and few-shot learning settings. This study contributes to the evolving landscape of health care informatics by showcasing the potential of advanced language models in extracting nuanced information critical for enhancing patient care.

**Methods:**

The main data source for analysis in this paper is Medical Information Mart for Intensive Care III data set. Among all notes in this data set, we focused on discharge summaries. Prompt engineering was undertaken, involving an iterative exploration of diverse prompts. Leveraging carefully curated examples and refined prompts, we investigate the model’s proficiency through zero-shot as well as few-shot prompting strategies.

**Results:**

Results show GPT’s varying effectiveness in identifying mentions of tobacco, alcohol, and substance use across learning scenarios. Zero-shot learning showed high accuracy in identifying substance use, whereas few-shot learning reduced accuracy but improved in identifying substance use status, enhancing recall and *F*_1_-score at the expense of lower precision.

**Conclusions:**

Excellence of zero-shot learning in precisely extracting text span mentioning substance use demonstrates its effectiveness in situations in which comprehensive recall is important. Conversely, few-shot learning offers advantages when accurately determining the status of substance use is the primary focus, even if it involves a trade-off in precision. The results contribute to enhancement of early detection and intervention strategies, tailor treatment plans with greater precision, and ultimately, contribute to a holistic understanding of patient health profiles. By integrating these artificial intelligence–driven methods into electronic health record systems, clinicians can gain immediate, comprehensive insights into substance use that results in shaping interventions that are not only timely but also more personalized and effective.

## Introduction

The use and misuse of psychoactive substances rank as critical risk elements for global health, contributing substantially to the worldwide disease burden [1,2]. Alcohol, tobacco, and illegal drugs are implicated in more than 80 identified conditions that lead to disease and injury [3,4], incurring significant health and societal costs [5-7]. Tobacco use is primarily connected to chronic diseases that often result in death, while alcohol consumption is associated with both acute conditions, such as injuries—both intentional and accidental—and chronic diseases, varying in mortality risk (eg, high risk includes liver cirrhosis and head and neck cancers; low risk covers conditions such as depression and alcohol dependency). Illicit drug use carries risks of infectious diseases, particularly through intravenous methods that may transmit HIV, in addition to heightened risks of suicide and drug use disorders. Unlike tobacco or illicit drugs, alcohol presents a complex profile, as certain levels and patterns of consumption have been shown to have protective effects against some diseases, notably coronary heart disease [8-10].

The documentation of substance use information in patient clinical notes plays an important role in care delivery by impacting clinical decision-making processes. First, it furnishes health care providers with vital information concerning a patient’s addiction history, a fundamental component in constructing a comprehensive medical profile [11]. This knowledge is instrumental in devising patient-centered treatment plans that not only address the primary medical concern but also consider the complexities of the use and its potential impact on treatment efficacy [12]. Furthermore, the extraction of this information aids in risk assessment, enabling the identification of patients who may be at higher risk of relapse or complications, thereby allowing for more proactive and tailored interventions [13]. The incorporation of extraction of substance use information from clinical notes directly informs patient treatment approaches. It enables health care providers to design interventions that address not only the immediate health concern but also the underlying addiction issue if exists [14]. The integration of substance use information into treatment planning facilitates the development of harm reduction strategies and medication-assisted therapies, tailored to each patient’s unique needs and readiness for change [15]. This patient-centered approach not only enhances treatment outcomes but also fosters a supportive therapeutic relationship, promoting long-term recovery and well-being. Ultimately, this information enhances the precision of clinical decision-making by fostering a holistic understanding of the patient’s health, thus underscoring the indispensability of addiction status extraction from clinical documentation in modern health care practice. By incorporating substance use information into risk assessment and treatment decision-making, health care professionals can deliver more precise, effective, and patient-centered care, identifying patients at higher risk of complications, relapse, or adverse outcomes due to their substance use history, ultimately leading to more targeted interventions and improved patient outcomes.

Studies [16,17] have used machine learning techniques to predict treatment outcomes for patients with substance use disorders, demonstrating how addiction status data can inform risk assessment and stratification. Studies [18,19] have explored risk stratification for opioid overdose, incorporating addiction status data and clinical information to identify patients at higher risk, thereby informing targeted interventions and care plans. Researchers also examine how addiction status information informs the development of personalized treatment plans and explore how health care providers tailor interventions to address both the primary medical issue and the underlying addiction concerns [20-22]. Many works emphasize the importance of patient-centered care and how extraction of substance addiction data enhances this approach. They highlight the significance of understanding a patient’s history of addiction for delivering more effective and empathetic care [23-28]. However, implications for health care policy and the implementation of substance use data into clinical practice have their own barriers and challenges [29-33].

The traditional process of extracting data related to substance use from clinical notes involves the use of rule-based approaches to parse the unstructured clinical narratives, identifying and categorizing relevant information pertaining to substance use. However, rule-based approaches lack a standardized rule language. On the other hand, the high variability in language found within clinical notes imposes significant limits on the accuracy of traditional techniques that rely on parsing rules to detect text patterns. Clinician typographical errors, abbreviations, and other linguistic variations hinder the effectiveness of these methods. Conversely, deep learning methods have shown impressive efficacy in extracting such information from the intricate and complex texts within clinical notes [34-36]. However, the necessity for extensive, high-quality annotated data sets for training—as information extraction is a supervised task in natural language processing—presents a significant challenge that must be overcome to fully realize the potential of these models in new and practical real-world settings.

Recently, large language models (LLMs) have emerged as a promising solution to this challenge, particularly due to their significant ability to “learn” and adapt to diverse language patterns without the need for additional model training [37]. LLMs demonstrate an unparalleled ability to comprehend nuances of clinical narratives, extracting meaning from diverse and complex medical texts. Although primarily trained on open-source and non–domain-specific texts, generative pretrained transformer (GPT) [38], as a recent development in LLMs, has underscored its effectiveness when applied to clinical notes [39,40]. GPT has also showcased its capability in US medical licensing examinations by achieving or even surpassing human-level performance in perception of clinical context [41]. This exceptional performance may be attributed to several factors, including the extensive model parameters, large pretraining data sets, and instruction tuning and optimization with reinforcement learning human feedback [42]. Recent works in the extraction of substance use information leverage LLMs such as Bidirectional Encoder Representations from Transformers and T5, with models being fine-tuned specifically for the social determinants of health extraction task [36,43]. The emergence of LLMs has also enabled new training paradigms, including few-shot or zero-shot learning [38,44].

Leveraging GPT (GPT-3.5 model), in this work we explore the extraction of patient’s substance use information in specific patients’ tobacco, alcohol, and illicit substance use information from their notes and assignment of a status to classifying the individual’s engagement with the substance into categories based on time-related factors (ie, past, present, or none). Since prompt engineering is essential when interacting with GPT to obtain high-quality responses, our proposed workflow involves performing zero-shot as well as few-shot prompting. In zero-shot learning, the model is expected to generalize to tasks without having seen any examples from that specific task during training. It relies on understanding the task description and applying previously learned knowledge and patterns to new, unseen situations. In a zero-shot learning scenario, the GPT is instructed to perform a particular task through an input prompt, and it produces text as a response, which serves as the output. For instance, when provided with the prompt: *List mentions of substance use in the following note: <clinical note>.* Then, the GPT extracts the reference to substance use with surrounding information relevant to it and produces the following output text—h/o prior tobacco abuse × 60 pack years—while few-shot learning involves training models on a very small data set. In this prompting setting, the model is designed to learn information from a few examples and generalize that knowledge to new data. We first experimented and formulated prompts to elicit the desired responses from the model. Then we used our finalized prompt in zero-shot learning setting. For few-shot prompting, we add a few examples to our finalized prompt to directly address the types of errors observed in zero-shot learning.

To the best of our knowledge, no scholarly publication has investigated the use of zero-shot and few-shot learning approaches with the GPT-3.5 model in the context of extracting data on patients’ substance use as well as determining their usage status. The substance use information is usually scattered throughout multiple clinical notes and may be overlooked by a new provider despite the fact that this information can affect clinical decision-making. By automating extraction of substance use profile from multiple clinical notes, the substance use status can be provided in a summarized format. It can also be used in automated clinical decision embedded into electronic health records (EHR). Secondary analysis of real-world data can be biased if it does not account for substance use profile. Automated extraction of substance use profiles can greatly facilitate generation of real-world evidence from EHR data. Our evaluation aims to provide insights into the capabilities and limitations of LLMs in substance use information extraction. Ultimately, our goal is to contribute to the ongoing development of the use of LLMs in the field of substance use information extraction, with the aim of improving the quality of care.

## Methods

### Study Design

The main data source for analysis in this paper is Medical Information Mart for Intensive Care III (MIMIC-III) data set. This data set is a widely used and comprehensive source of deidentified health care data. It contains detailed clinical information from more than 60,000 critical care patients admitted to the Beth Israel Deaconess Medical Center in Boston, Massachusetts, spanning a period of nearly a decade. This rich data set includes EHR, laboratory results, prescription records, and clinical notes, making it a valuable resource for medical research, particularly in the fields of critical care, epidemiology, and health informatics. Among all notes in this data set, we focused on discharge summaries. These notes typically provide a comprehensive overview of a patient’s hospital stay, including the reason for admission, the treatments and procedures performed, and the patient’s medical and social history and recommendations for postdischarge care. The social history section of a discharge summary typically covers various aspects of patients’ life, such as their marital status, living situation, occupation, and lifestyle factors. If a patient has a history of addiction, particularly if it is relevant to the reason for his or her hospitalization or has implications for his or her postdischarge care, it is included in this section. The MIMIC-III contains 59,652 discharge summaries of 46,146 patients, among which we selected the patients with history of chronic obstructive pulmonary disease (COPD). Most of the patients with COPD are represented by older adults for whom identification of substance use information plays an important role in establishing an optimal treatment plan. Patients with COPD often have a history of smoking, as cigarette smoking is a primary risk factor for the development of COPD. Many individuals diagnosed with COPD have a significant smoking history. Alcohol and drug addiction are not typically considered direct risk factors for the development of COPD. However, substance abuse can exacerbate COPD symptoms, hinder treatment compliance, and lead to a more rapid decline in lung function in individuals already diagnosed with the disease [45-49].

Among 1646 patients with COPD, we selected discharge summary for 500 random patients, which was shown sufficient for assessing natural language processing pipeline accuracy in previous studies [50-53]. In this study, we use GPT and in specific GPT-3.5 model for generative question answering. We leveraged most capable and most cost-effective model in the GPT-3.5 family, which is GPT-3.5-turbo. This model has been optimized for chat. We accessed this model through the chat completions Application Programming Interface end point for extraction of substance use information, in specific patient’s tobacco, alcohol, and illicit substance use, in 2 learning settings: zero-shot and few-shot.

In the zero-shot learning setting, a model is presented with tasks or queries for which it has not received explicit training. It is expected to extrapolate knowledge from its preexisting understanding of language and context to generate meaningful responses. This setting challenges the model to generalize effectively and showcase adaptability to novel prompts, reflecting its capacity to comprehend and manipulate language beyond the scope of its training data.

On the contrary, few-shot learning involves training a model on a minimal number of task-specific examples. In this setting, GPT is provided with a few examples, allowing it to learn task-specific patterns and nuances. This approach leverages the model’s pretrained knowledge to swiftly adapt to new tasks, demonstrating a remarkable capability for transfer learning. Few-shot learning is particularly advantageous when dealing with tasks that require a prompt-specific understanding, as it empowers the model to distill essential information from a handful of examples and apply this knowledge to generate coherent and contextually relevant responses.

Since prompt engineering is essential when interacting with GPT to obtain high-quality responses, we first experimented and formulated prompts to elicit the desired responses from the model. Then we used our finalized prompt in zero-shot learning setting. Multimedia Appendix 1 shows examples of our examined prompts, and Multimedia Appendix 2 shows our finalized prompt along with GPT responses to this prompt on different notes. These multimedia appendices show that we experimented different prompts and our finalized prompt was selected to be as follows:

Using the following patient’s text, list tobacco use, illicit substance use, and alcohol use mentions and each one's status (“present,” “past,” and “none”) in the bullets: <clinical note>.

The reason for selecting this prompt is that it provides the most comprehensive and detailed guidance for the task compared with the other prompts. This prompt is the only one that provides a clear and specific set of instructions. It not only asks to list data of tobacco, illicit substances, and alcohol use but also instructs GPT to include the status of usage as being “present,” “past,” or “none.” This specificity helps guide the model to provide a more detailed and informative response. This prompt, in addition, is well structured and unambiguous in its request. It leaves no room for interpretation regarding what information is expected, making it easier for the model to generate accurate and relevant content. Moreover, in the context of medical or health care–related information, knowing the status of use (whether it is current, past, or not present) is critical for patient care and understanding their health history. This prompt includes this essential aspect, making it the most informative and complete prompt. While the last prompt in Multimedia Appendix 1 is also relatively detailed, it does not specify the need to provide the usage status for each category, which can be a crucial element in a medical or clinical context.

Next, we conducted error analysis that involves a detailed examination of the model’s outputs to find specific instances where the model is underperforming. This process helps in selecting the most instructive examples to be included for few-shot learning in order to improve the model’s performance. Multimedia Appendix 3 shows instances in which GPT underperformed. Considering first text, it can be seen that GPT had errors on assigning the use status of “None” to all type of substances. The clinical text indicates “No hx of tobacco or EtOH,” where hx stands for history. The phrase “No hx” explicitly indicates that there is no history, which should correspond to the status of “none” for both tobacco and alcohol use. GPT’s output failed to recognize this nuance. It interpreted “No hx” as a lack of mention, rather than an absence of use, and did not assign a status as instructed. GPT, like many language models, relies heavily on context to make predictions. Without a more extensive context, it might be challenging for the model to deduce that “no history of tobacco” implies “None” as tobacco use status without specific training or instructions. On the other hand, the use of negative phrasing, such as “no hx of,” can be challenging for models to interpret correctly, especially without specialized training. Moreover, in shorter phrases or isolated sentences, the model may not have enough context to accurately infer the intended meaning. Furthermore, GPT’s response “No mention of illicit substance use” suggests that there was no information provided about illicit substances, which is a correct extraction but lacks the explicit assignment of none status.

The second clinical note states: “No h/o tobacco and rare Etoh, no IVDA.” Here, h/o stands for history of, Etoh stands for ethyl alcohol, and IVDA stands for intravenous drug abuse. Investigating GPT’s response on this text shows that GPT’s response was partially incorrect. While it did correctly identify that there is “None” for tobacco use, it failed to echo the specific language of the note, which included “No h/o tobacco,” with h/o meaning “history of.” In clinical contexts, maintaining the specific terminology used in patient records is crucial for accuracy and clarity.

Similarly, for illicit substance use, GPT’s response of “None” is correct in the absence of use but lacks the explicit mention of “no IVDA” found in the clinical note. For alcohol use, the phrase “rare Etoh” suggests infrequent but current use of alcohol. The correct status should be “present” since it implies ongoing use. GPT’s output incorrectly marked this as “past,” which is an error. The phrase “rare” does not indicate cessation of use but rather infrequency and should be understood within the current context unless historical context is provided to imply past use.

Investigating GPT’s response on third text also shows GPT’s inability to correctly identify “Denies alcohol/drugs” as “None” for alcohol and illicit substance use. The phrase “Denies alcohol/drugs” is linguistically complex, and the model may not easily interpret its negation. In addressing the identified discrepancies within the model’s output, it is imperative to rectify the inaccuracies by aligning the generated responses with the precise medical terminology and context presented in the clinical notes. The process entails reformulating the outputs to accurately reflect the specific language used, such as “No h/o tobacco” to denote a nonhistory of tobacco use. The refined examples, embodying both the exact phrasing and the proper status assignments, should then be systematically integrated into the training regime of the model through few-shot learning. This integration will facilitate the model’s proficiency in comprehending and processing medical shorthand and context-sensitive information, thereby enhancing its performance on tasks that involve the extraction of nuanced data from clinical documentation. Through iterative exposure to these corrected instances, the model will incrementally improve its ability to discern and categorize substance use information with a higher degree of accuracy and reliability, a crucial aspect for applications within clinical settings. Finally, Multimedia Appendix 4 shows the edited prompt for few-shot learning.

### Ethical Considerations

No protected health information was collected, and the analytical data set was fully de-identified. To process the data, HIPAA (Health Insurance Portability and Accountability Act)-compliant Microsoft Azure OpenAI Application Programming Interface has been used.

## Results

Among the 59,625 discharge summaries included in the MIMIC data set, 2043 were specifically associated with patients having a history of COPD. These particular summaries corresponded to a total of 1646 distinct patients with COPD. From this cohort, a random selection process was applied to obtain discharge summaries for a subset of 500 individuals for further analysis.

Table 1 presents general statistics pertaining to the data set. This table provides demographic information, presenting the distribution of attributes among the surveyed population. The data include the percentage breakdown of individuals based on gender, ethnicity, and marital status. The gender distribution shows a relatively balanced representation, with 53% male and 47% female respondents. This suggests a fair inclusion of both genders in the study. The majority of the surveyed population identifies as “White,” constituting 73.16%. “Black,” “Asian,” and “Other” ethnicities make up 11.69%, 1.52%, and 13.63%, respectively. The marital status distribution reveals that a significant portion of the respondents is married (43.07%), followed by widowed (23.81%) and single (22.29%) individuals. There is also a small percentage with unknown marital status (4.98%), and divorced (4.55%) and separated (1.30%) individuals make up the rest. Accuracy, precession, recall, and *F*_1_-score have been selected as evaluation metrics noting that every metrics have been calculated by manually reviewing all notes in data set.

Table 2 provides an overview of the results obtained from using GPT for the extraction of substance-related mentions and the corresponding status of the usage, comparing few-shot learning and zero-shot learning settings across tobacco, drug, and alcohol categories. The noticeable discrepancy between the accuracy of substance use mentions and status extraction in the zero-shot setting suggests a potential area for improvement, particularly in the nuanced understanding of the status associated with all categories of substance use. To leverage this insight and transition toward few-shot learning, we examined the specific instances where the zero-shot model struggled to accurately extract usage statuses and identified patterns, types of sentences, or contextual cues that may had contributed to the lower accuracy in extraction. Multimedia Appendix 3 shows multiple instances on which GPT made errors. These instances were used to update the finalized prompt for few-shot learning. Multimedia Appendix 4 shows our finalized prompt for few-shot learning.

**Table 1:** General statistics.

Attributes		Proportion, %
**Sex**		
	Female	47
	Male	53
**Ethnicity**		
	White	73.16
	Black	11.69
	Asian	1.52
	Other	13.63
**Marital status**		
	Married	43.07
	Widowed	23.81
	Single	22.29
	Unknown	4.98
	Divorced	4.55
	Separated	1.30

**Table 2:** Performance of generative pretrained transformer in a zero-shot and few-shot learning setting.

		Few-shot learning		Zero-shot learning	
		Mention (%)	Status (%)	Mention (%)	Status (%)
**Tobacco**					
	Recall	87	66	93	29
	Precision	58	51	98	87
	*F*_1_-score	70	57.5	96	43.5
	Accuracy	60	40	92	26
**Drug**					
	Recall	88	89	92	34
	Precision	93	89	99	100
	*F*_1_-score	91	89	95	51
	Accuracy	82	79	90	32
**Alcohol**					
	Recall	89	78	89	29
	Precision	78	73	99	100
	*F*_1_-score	83	76	94	45
	Accuracy	71	57	90	29

In zero-shot learning, for tobacco, the precision was at 98%, and for both drug and alcohol mentions, it reached an impressive 99%. The recall for tobacco mentions was 93%, suggesting that the model was able to identify a large majority of the relevant instances. However, the recall for the status of tobacco use was substantially lower at 29%. For drugs, the recall was also high at 92% for mentions but significantly lower at 34% for the status. Similarly, for alcohol, the recall was 89% for mentions but dropped to 29% for the status. The *F*_1_-scores, which balance recall and precision, were quite high for mentions, with tobacco at 96%, drugs at 95%, and alcohol at 94%, indicating strong overall performance in this aspect. Nevertheless, the *F*_1_-scores for the status were lower: 43.5% for tobacco, 51% for drugs, and 45% for alcohol.

After few-shot learning, the accuracy of extraction of status was changed from 26%, 32%, and 29% to 40%, 79%, and 57%, for tobacco, alcohol, and substance use, respectively. The observed changes in the accuracy of extraction of status of the usage, following the incorporation of a new crafted prompt and the inclusion of examples where GPT previously had errors, indicate a 14%, 47%, and 28% improvement in the model’s performance in terms of tobacco, drug, and alcohol status use extraction, respectively. On the other hand, few-shot learning led to significant decrease in the accuracy of mentions of substance use across all categories. The accuracy of extraction of tobacco, alcohol, and substance use mentions in zero-shot setting scenario was 92%, 90%, and 90%, respectively. While the accuracy for the use mentions in few-shot setting was 60%, 82%, and 71%, respectively.

Regarding the extraction of mentions of substance use, in few-shot learning, for tobacco, the recall is high at 87%, but precision is comparatively lower at 58%, resulting in a balanced *F*_1_-score of 70%. Similar patterns are observed for alcohol category. While in contrast, precision value for mentions of drug use (93%) is higher than recall value (88%). Zero-shot learning exhibits higher recall in extraction of use mentions for all substance use categories, ranging from 89% to 93%, with precision ranging from 98% to 99%. Consequently, *F*_1_-scores vary between 94% and 96%.

Regarding the extraction of the usage status, in few-shot learning, the recall value for tobacco is 66%, with precision just more than 50% and *F*_1_-score of 57.5%. While in comparison with few-shot learning, zero-shot learning resulted in 37% lower recall and 14% lower *F*_1_-score but 36% higher precision. The same pattern can be seen for alcohol and drug use status extraction across both learning setting, meaning lower recall and higher precision in zero-shot learning compared with few-shot learning resulted in higher *F*_1_-score in few-shot learning. The discrepancies observed in the extraction performance metrics before and after few-shot learning may be attributed to several factors related to model configuration, prompt specificity, and data characteristics. First, the model configuration in few-shot learning involves exposure to specific examples that may not be diverse enough, potentially leading the model to overfit to particular features of the examples provided rather than generalizing effectively. This overfitting could result in reduced precision in mention extraction as the model becomes more sensitive to the nuances of the few-shot examples at the cost of broader applicability.

Second, prompt specificity plays a significant role in directing the model’s attention and interpretation mechanisms. In the few-shot scenario, if the prompts are crafted with high specificity toward the status of use, the model’s focus might shift from mention detection toward status classification, explaining the improvement in status extraction accuracy and the concomitant decline in mention extraction accuracy.

Finally, data characteristics, such as the complexity, ambiguity, and representativeness of the clinical notes, can significantly influence the outcomes. Few-shot learning might result in better recall for status extraction if the examples chosen for training closely resemble the test cases, indicating that these examples were well selected to represent the variety of ways that status can be expressed in clinical texts. Conversely, zero-shot learning’s higher precision suggests that the model, without the bias of the few-shot examples, might be more conservative and specific in its outputs, thus avoiding false positives.

## Discussion

### Principal Findings

The process of trying diverse prompts and selecting the one that yields the desired output was instrumental in harnessing the capabilities of GPT to align with objectives of this study. The act of crafting varied prompts allowed to explore the model’s versatility and adaptability in correct extraction of patients’ substance usage status. By experimenting with different prompt formulations, it becomes feasible to ascertain the prompt’s impact on the model’s behavior, leading to high accuracy in extraction. The finalized prompt in zero-shot is well structured and unambiguous in its request. It leaves no room for interpretation regarding what information is expected, making it easier for the model to generate accurate and relevant content. Moreover, in the context of medical or health care–related information, knowing the status of substance use (whether it is current, past, or none) is critical for patient care and understanding their health history. This prompt includes this essential aspect, making it the most informative and complete prompt. While the last prompt in Multimedia Appendix 1 is also relatively detailed, it does not specify the need to provide the status of each mention, which can be a crucial element in a medical or clinical context.

Crafting the new prompt by strategically using few-shot learning and tailoring to the challenges observed in the zero-shot setting resulted in increase on the accuracy of extraction of usage status. This approach capitalizes the importance of providing targeted guidance to enhance the model’s proficiency in extracting nuanced information related to tobacco, alcohol, and substance uses. While the progress is commendable, it is essential to recognize that model refinement is an iterative process. Continued iterations, incorporating additional examples and refining the prompt, may further enhance accuracy, particularly in scenarios with inherent complexities.

The presented results in Table 2 highlight the contrasting performance of GPT in extracting mentions of tobacco, alcohol, and substance use in both zero-shot and few-shot learning scenarios. In the zero-shot setting, the accuracy for extraction of tobacco, alcohol, and substance use mentions is notably high. However, in the few-shot setting, the accuracy diminishes significantly. On the contrary, few-shot learning led to significant increase in devising the status of substance use compared with zero-shot learning (significant increase in recall and *F*_1_-score). However, this improvement comes at the cost of a reduction in precision in both substance use information extraction and devising the status of the use. Accordingly, the selection between zero-shot and few-shot learning hinges on the goals of the task. Zero-shot learning excels in precisely extracting use mentions, demonstrating its effectiveness in situations in which comprehensive recall is paramount. Conversely, few-shot learning offers advantages when accurately determining the status of use is the primary focus, even if it involves a trade-off in precision.

The models we developed can be integrated with EHR systems to automatically extract and update patient substance use information. This integration facilitates real-time updates to patient profiles, ensuring that health care providers have access to the most current data when making treatment decisions. By embedding our models into clinical decision support systems, health care providers can receive proactive alerts and recommendations based on the extracted data. For example, if a patient’s history of substance use changes, the system could automatically suggest modifications to his or her treatment plan or recommend additional screenings.

In acknowledging the limitations of this study, it is important to recognize the constraints imposed by the use of a single model, GPT-3.5, which, while demonstrating substantial capabilities, also exhibits specific challenges in processing complex linguistic structures such as negations and subtle context cues. This limitation notably impacted the accuracy of status identification in zero-shot learning settings, where the model sometimes failed to correctly interpret negations, leading to errors in status assignment.

Furthermore, the study’s reliance on the MIMIC-III data set, while extensive, limits the generalizability of findings across diverse demographic and clinical settings. The data set’s inherent biases and the specific clinical environment from which it was derived might not fully represent the broader patient populations encountered in different geographic or health care contexts. To address these limitations, future research should consider using a multimodel approach to validate findings and enhance the robustness of the conclusions drawn. Incorporating additional models such as Bidirectional Encoder Representations from Transformers may provide comparative insights and help mitigate the biases of a single model approach. Moreover, expanding the data set to include a wider array of clinical environments and patient demographics would enhance the generalizability of the artificial intelligence tools developed. In addition, the implementation of advanced training techniques, including more sophisticated prompt engineering and error analysis methodologies, could further refine the artificial intelligence’s understanding of complex clinical narratives.

### Conclusion and Future Work

The extraction of psychoactive substance use status from clinical notes holds significant implications for risk assessment and patient treatment. It empowers health care providers to perform risk evaluations and to devise individualized treatment plans, leading to enhancing the precision and efficacy of care delivery while addressing the complex interplay between medical conditions and addiction. In this study, we investigate the efficacy of 2 prompt-based approaches—zero-shot and few-shot learning—for extracting patient’s substance use information from discharge summaries of patients with COPD using GPT-3. Our findings indicate that GPT-3’s few-shot learning capabilities serve as a promising starting point for extracting status of substance use without the need for annotated data. The GPT-3 exhibited high precision but lower recall, suggesting a conservative approach that yields fewer false positives but may miss relevant cases. Conversely, few-shot learning demonstrated a marked improvement in recall, indicating a greater ability to identify relevant instances, yet at the expense of precision. The implications of these findings are significant for the landscape of clinical practice, where the accurate assessment of usage status is crucial for risk assessment and tailoring patient treatment plans. The enhanced recall in few-shot learning suggests its use in scenarios where missing a case of substance use is highly detrimental, while the high precision of zero-shot learning would be preferred in contexts where the cost of false positives is greater. Therefore, researchers and practitioners should carefully consider the emphasis on recall, precision, and the overall balance between the 2 when deciding between these learning scenarios based on the specific requirements of their application. We prompted GPT-3 with only 4 randomly selected samples. More examples for few-shot learning may improve the performance. In addition, our reliance on the MIMIC-III data set, though comprehensive, restricts the generalizability of our findings. The data set’s inherent biases and its derivation from a specific clinical environment may not accurately reflect the varied patient populations found across different geographic or health care settings. Despite these limitations, the study presents a significant step forward in our understanding of the capabilities and limitations of advanced language models in the critical domain of health care. As the future work, we investigate the capability of LLMs in extraction of the quantity, frequency, duration, and severity of substance use disorder.

## Appendix

Multimedia Appendix 1 Examples of 3 different prompts to GPT-3.5 and its responses.

Multimedia Appendix 2 Finalized prompt and examples of 3 GPT-3.5 responses to this prompt.

Multimedia Appendix 3 Examples on which generative pretrained transformer had errors.

Multimedia Appendix 4 Crafted prompt for few-shot learning setting.

## Abbreviations

COPD: chronic obstructive pulmonary disease
EHR: electronic health record
GPT: generative pretrained transformer
HIPAA: Health Insurance Portability and Accountability Act
LLM: large language model
MIMIC-III: Medical Information Mart for Intensive Care III

## References

[1] Ezzati M, Lopez AD, Rodgers A, Murray CJL (2004). Comparative quantification of health risks. Global and Regional Burden of Disease Attributable to Selected Major Risk Factors.

[2] Rehm J, Room R (2005). The global burden of disease attributable to alcohol, tobacco and illicit drugs. Preventing Harmful Substance Use: The Evidence Base for Policy and Practice.

[3] Rehm J, Patra J, Popova S (2006). Alcohol-attributable mortality and potential years of life lost in Canada 2001: implications for prevention and policy. Addiction.

[4] Popova S, Rehm J, Patra J (2018). Illegal drug-attributable mortality and potential years of life lost in Canada 2002: implications for prevention and policy. Contemp Drug Probl.

[5] Single E, Robson L, Xie X, Rehm J (1998). The economic costs of alcohol, tobacco and illicit drugs in Canada, 1992. Addiction.

[6] Andlin-Sobocki P (2004). Economic evidence in addiction: a review. Eur J Health Econ.

[7] Andlin-Sobocki P, Rehm J (2005). Cost of addiction in Europe. Eur J Neurol.

[8] Rehm J, Room R, Graham K, Monteiro M, Gmel G, Sempos CT (2003). The relationship of average volume of alcohol consumption and patterns of drinking to burden of disease: an overview. Addiction.

[9] Rehm J, Sempos CT, Trevisan M (2003). Average volume of alcohol consumption, patterns of drinking and risk of coronary heart disease—a review. Eur J Cardiovas Prev Rehabil.

[10] Corrao G, Rubbiati L, Bagnardi V, Zambon A, Poikolainen K (2000). Alcohol and coronary heart disease: a meta-analysis. Addiction.

[11] Volkow ND (2010). Drugs, Brains, and Behavior: The Science of Addiction.

[12] McLellan AT, Lewis DC, O'Brien CP, Kleber HD (2000). Drug dependence, a chronic medical illness: implications for treatment, insurance, and outcomes evaluation. JAMA.

[13] Bogenschutz MP, Donovan DM, Mandler RN, Perl HI, Forcehimes AA, Crandall C, Lindblad R, Oden NL, Sharma G, Metsch L, Lyons MS, McCormack R, Konstantopoulos WM, Douaihy A (2014). Brief intervention for patients with problematic drug use presenting in emergency departments. JAMA Intern Med.

[14] Babor TF, McRee BG, Kassebaum PA, Grimaldi PL, Ahmed K, Bray J (2007). Screening, brief intervention, and referral to treatment (SBIRT): toward a public health approach to the management of substance abuse. Subst Abus.

[15] Minozzi S, Amato L, Bellisario C, Davoli M (2014). Maintenance treatments for opiate-dependent adolescents. Cochrane Database Syst Rev.

[16] Acion L, Kelmansky D, van der Laan M, Sahker E, Jones D, Arndt S (2017). Use of a machine learning framework to predict substance use disorder treatment success. PLoS One.

[17] Tapia-Galisteo J, Iniesta JM, Perez-Gandia C, Garcia-Saez G, Puertolas DU, Izquierdo FJ, Hernando ME (2020). Prediction of cocaine inpatient treatment success using machine learning on high-dimensional heterogeneous data. IEEE Access.

[18] Weiner SG, Baker O, Bernson D, Schuur JD (2020). One-Year mortality of patients after emergency department treatment for nonfatal opioid overdose. Ann Emerg Med.

[19] Shah-Mohammadi F, Cui W, Bachi K, Hurd Y, Finkelstein J (2022). Using natural language processing of clinical notes to predict outcomes of opioid treatment program.

[20] Rich KM, Bia J, Altice FL, Feinberg J (2018). Integrated models of care for individuals with opioid use disorder: how do we prevent HIV and HCV?. Curr HIV/AIDS Rep.

[21] Schwartz RP, Kelly SM, Mitchell SG, Gryczynski J, O'Grady KE, Gandhi D, Olsen Y, Jaffe JH (2017). Patient-centered methadone treatment: a randomized clinical trial. Addiction.

[22] Englander H, Dobbertin K, Lind BK, Nicolaidis C, Graven P, Dorfman C, Korthuis PT (2019). Inpatient addiction medicine consultation and post-hospital substance use disorder treatment engagement: a propensity-matched analysis. J Gen Intern Med.

[23] Miller D, Steele Gray C, Kuluski K, Cott C (2015). Patient-centered care and patient-reported measures: let's look before we leap. Patient.

[24] Novilla MLB, Goates MC, Leffler T, Novilla NKB, Wu C, Dall A, Hansen C (2023). Integrating social care into healthcare: a review on applying the social determinants of health in clinical settings. Int J Environ Res Public Health.

[25] Strauss T (2020). Organizational factors underlying the adoption of a patient-centered approach in physician-patient interaction [dissertation]. Israel: University of Haifa.

[26] Jones KG, Roth SE, Vartanian KB (2022). Health and health care use strongly associated with cumulative burden of social determinants of health. Popul Health Manag.

[27] Karapareddy V (2019). A review of integrated care for concurrent disorders: cost effectiveness and clinical outcomes. J Dual Diagn.

[28] King C, Collins D, Patten A, Nicolaidis C, Englander H (2022). Trust in hospital physicians among patients with substance use disorder referred to an addiction consult service: a mixed-methods study. J Addict Med.

[29] Farhud DD, Zokaei S (2021). Ethical issues of artificial intelligence in medicine and healthcare. Iran J Public Health.

[30] Rothstein MA (2007). Health privacy in the electronic age. J Leg Med.

[31] Price WN, Cohen IG (2019). Privacy in the age of medical big data. Nat Med.

[32] Crowley RA, Kirschner N, Health and Public Policy Committee of the American College of Physicians (2015). The integration of care for mental health, substance abuse, and other behavioral health conditions into primary care: executive summary of an American College of Physicians position paper. Ann Intern Med.

[33] Edmunds M, Frank R, Hogan M, McCarty D, Robinson-Beale R, Weisner C (1997). Managing Managed Care: Quality Improvement in Behavioral Health.

[34] Poulsen MN, Freda PJ, Troiani V, Davoudi A, Mowery DL (2022). Classifying characteristics of opioid use disorder from hospital discharge summaries using natural language processing. Front Public Health.

[35] Patra B, Sharma M, Vekaria V, Adekkanattu P, Patterson O, Glicksberg B, Lepow LA, Ryu E, Biernacka JM, Furmanchuk A, George TJ, Hogan W, Wu Y, Yang X, Bian J, Weissman M, Wickramaratne P, Mann JJ, Olfson M, Campion TR, Weiner M, Pathak J (2021). Extracting social determinants of health from electronic health records using natural language processing: a systematic review. J Am Med Inform Assoc.

[36] Romanowski B, Ben Abacha A, Fan Y (2023). Extracting social determinants of health from clinical note text with classification and sequence-to-sequence approaches. J Am Med Inform Assoc.

[37] Zhao WX, Zhou K, Li J, Tang T, Wang X, Hou Y, Min Y, Zhang B, Zhang J, Dong Z, Du Y, Yang C, Chen Y, Chen Z, Jiang J, Ren R, Li Y, Tang X, Liu Z, Liu P, Nie J, Wen J A survey of large language models. arXiv.

[38] (2023). GPT-4 technical report. OpenAI.

[39] Zhou J, Li T, Fong SJ, Dey N, González-Crespo R (2023). Exploring ChatGPT's potential for consultation, recommendations and report diagnosis: gastric cancer and gastroscopy reports’ case. Int J Interact Multimed Artif Intell.

[40] Choi HS, Song JY, Shin KH, Chang JH, Jang B (2023). Developing prompts from large language model for extracting clinical information from pathology and ultrasound reports in breast cancer. Radiat Oncol J.

[41] Singhal K, Azizi S, Tu T, Mahdavi SS, Wei J, Chung HW, Scales N, Tanwani A, Cole-Lewis H, Pfohl S, Payne P, Seneviratne M, Gamble P, Kelly C, Babiker A, Schärli N, Chowdhery A, Mansfield P, Demner-Fushman D, Agüera Y Arcas B, Webster D, Corrado GS, Matias Y, Chou K, Gottweis J, Tomasev N, Liu Y, Rajkomar A, Barral J, Semturs C, Karthikesalingam A, Natarajan V (2023). Large language models encode clinical knowledge. Nature.

[42] Ouyang L, Wu J, Jiang X, Almeida D, Wainwright C, Mishkin P, Zhang C, Agarwal S, Slama K, Ray A, Schulman J (2022). Training language models to follow instructions with human feedback. Adv Neural Inf Process Syst.

[43] Lybarger K, Ostendorf M, Yetisgen M (2021). Annotating social determinants of health using active learning, and characterizing determinants using neural event extraction. J Biomed Inform.

[44] Brown T, Mann B, Ryder N, Subbiah M, Kaplan JD, Dhariwal P, Neelakantan A, Shyam P, Sastry G, Askell A, Agarwal S (2020). Language models are few-shot learners. Adv Neural Inf Process Syst.

[45] Saeed AM, Raafat RH, Muneer MM (2023). Study of addiction in COPD patients in abbassia chest hospital. QJM Int J Med.

[46] Mahmoud EM, Mohammed ZA, El hawary AE, Ibrahim DA (2023). Screening for drug misusers in exacerbated chronic obstructive pulmonary disease (COPD) patients. Med Updates.

[47] Rabe KF, Hurd S, Anzueto A, Barnes PJ, Buist SA, Calverley P, Fukuchi Y, Jenkins C, Rodriguez-Roisin R, van Weel C, Zielinski J, Global Initiative for Chronic Obstructive Lung Disease (2007). Global strategy for the diagnosis, management, and prevention of chronic obstructive pulmonary disease: GOLD executive summary. Am J Respir Crit Care Med.

[48] Carlin B (2007). Chronic obstructive pulmonary disease: "learn more, breathe better". J Cardiopulm Rehabil Prev.

[49] American Lung Association Learn about COPD.

[50] Shah-Mohammadi F, Finkelstein J (2024). NLP-assisted differential diagnosis of chronic obstructive pulmonary disease exacerbation. Stud Health Technol Inform.

[51] Cui W, Shah-Mohammadi F, Finkelstein J (2023). Using electronic medical records and clinical notes to predict the outcome of opioid treatment program. Stud Health Technol Inform.

[52] Wang Y, Chen ES, Pakhomov S, Arsoniadis E, Carter EW, Lindemann E, Sarkar IN, Melton GB (2015). Automated extraction of substance use information from clinical texts. AMIA Annu Symp Proc.

[53] Shah-Mohammadi F, Cui W, Finkelstein J (2021). Entity extraction for clinical notes, a comparison between metamap and amazon comprehend medical. Stud Health Technol Inform.

