# Supplementary figures and images for: Extraction of Substance Use Information From Clinical Notes: Generative Pretrained Transformer–Based Investigation
Source: JMIR Med Inform. 2024 Aug 19;12:e56243. doi: 10.2196/56243 (PMC11369538; doi:10.2196/56243)

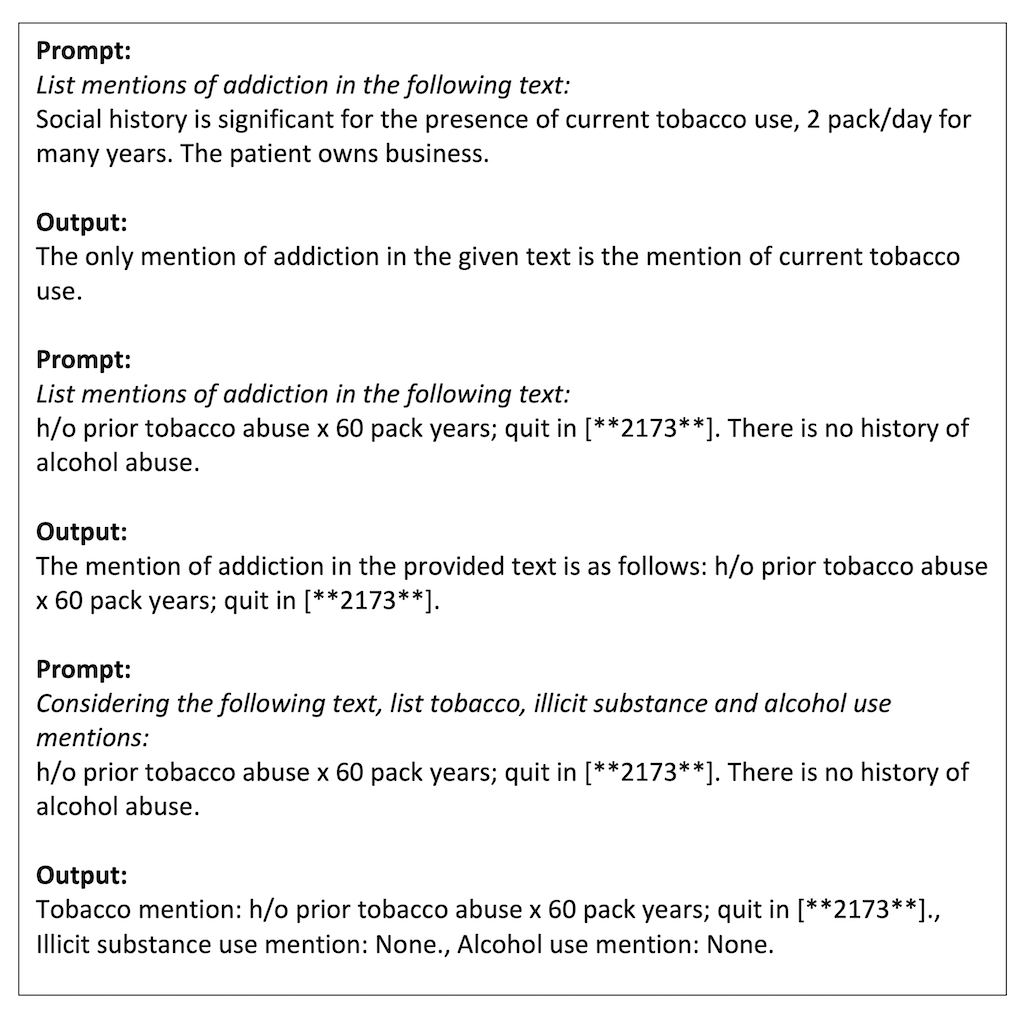

Supplement: Multimedia Appendix 1 [file medinform_v12i1e56243_app1.png]

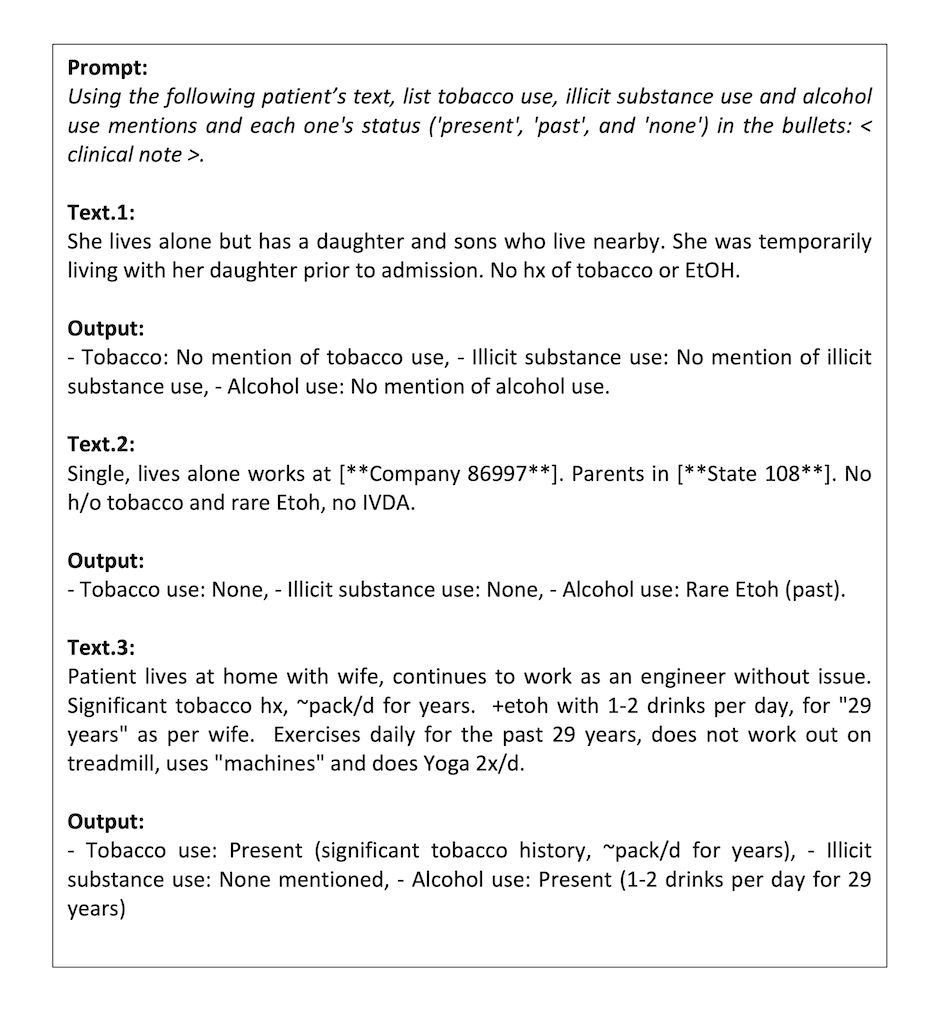

Supplement: Multimedia Appendix 2 [file medinform_v12i1e56243_app2.png]

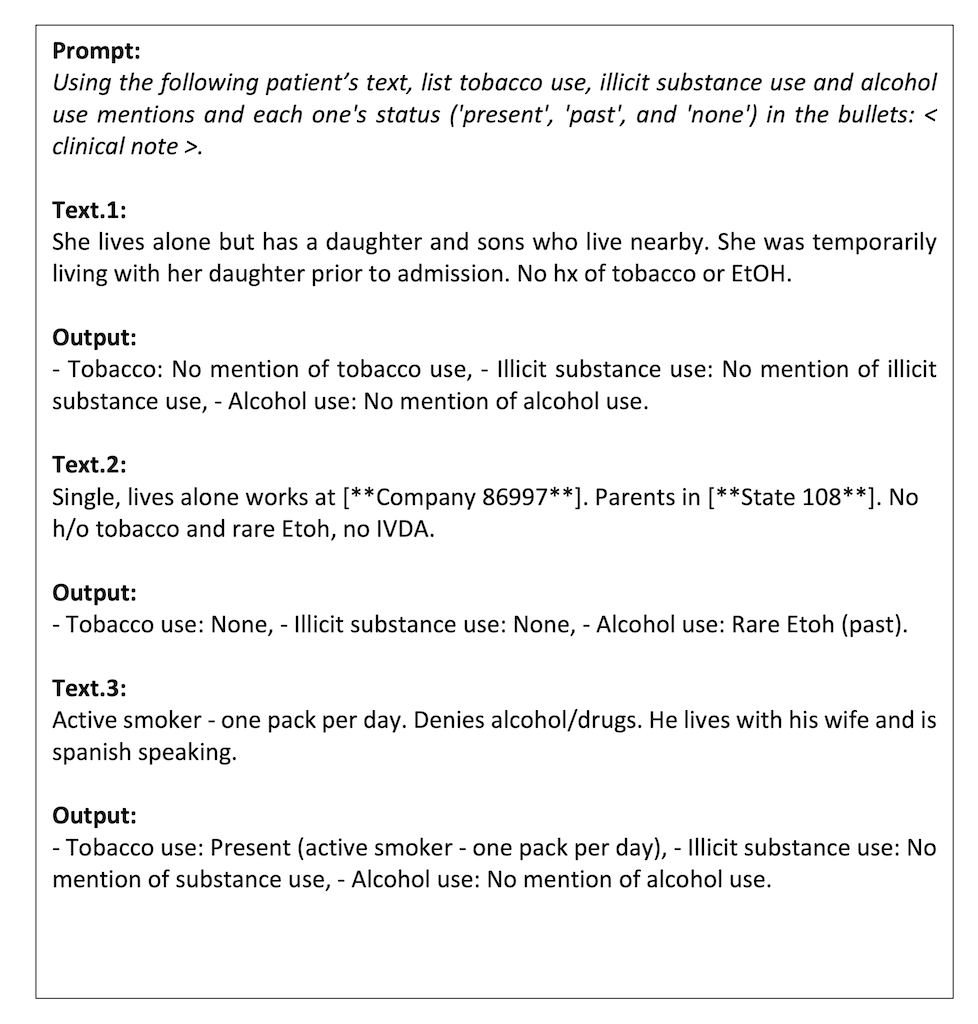

Supplement: Multimedia Appendix 3 [file medinform_v12i1e56243_app3.png]

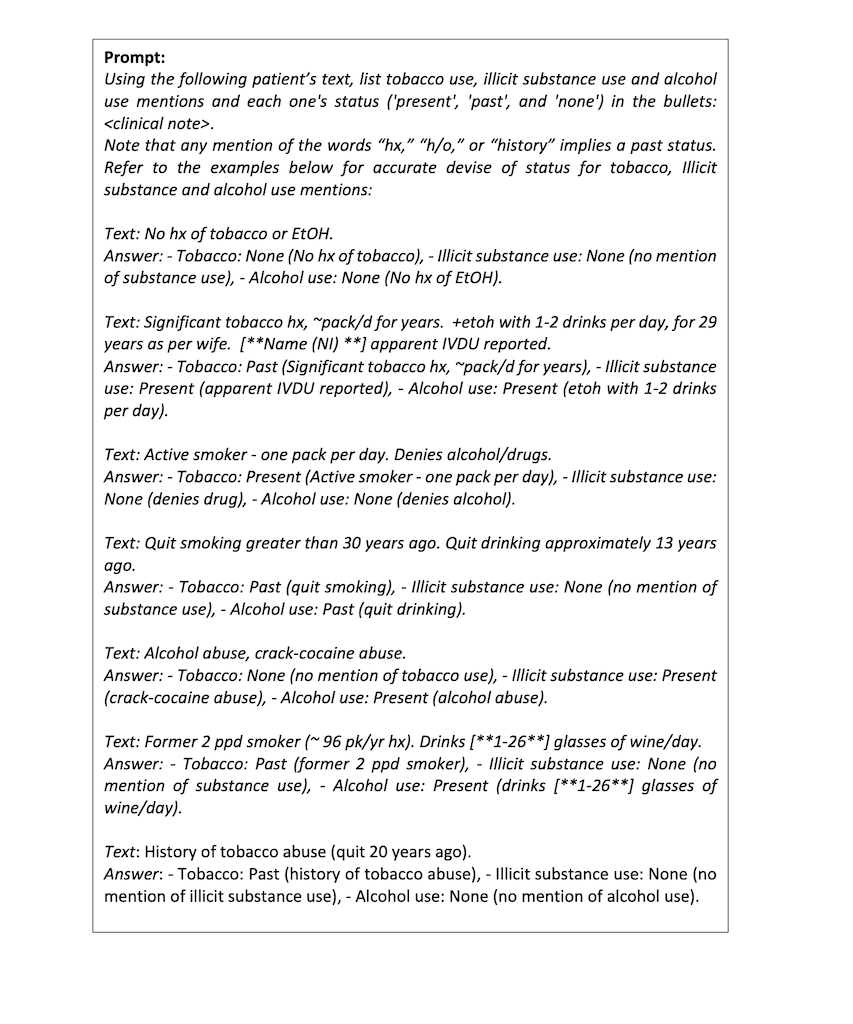

Supplement: Multimedia Appendix 4 [file medinform_v12i1e56243_app4.png]
